# Multidimensional ST Injury-State Dynamics During Controlled Human Coronary Occlusion and Balloon Deflation: An Analysis of the STAFF III Database

**DOI:** 10.7759/cureus.117811

**Published:** 2026-10-12

**Authors:** Patrick Dumitrescu, Johnny Cobas Hernandez, Renato Gustavo Luque, Igor Antonio Narciso

**Affiliations:** 1 Internal Medicine, LeeHealth, Florida State University College of Medicine, Cape Coral, USA

**Keywords:** coronary occlusion, electrocardiography, myocardial ischemia, st segment, vectorcardiography

## Abstract

Background: The 12-lead electrocardiogram (ECG) is central to the evaluation of acute coronary syndromes, but conventional ST metrics compress a multilead, time-varying response into a small number of scalar amplitudes. We developed a multidimensional ST injury-state framework to quantify the magnitude, organization, and temporal evolution of the ischemic ECG response. Materials and methods: We performed a secondary analysis of 152 controlled balloon occlusions in 104 patients from the STAFF III database. The principal territory analysis included 149 occlusions in 102 patients with left anterior descending (LAD), right coronary artery (RCA), or left circumflex (LCx) occlusion. Using overlapping 10-second windows, we constructed a baseline-adjusted 6 × 7 ST injury surface (leads V1-V6 at R + 80 to R + 200 ms) and an eight-dimensional ST injury vector (leads I, II, and V1-V6). The primary outcomes were peak injury-surface burden and peak vector magnitude. Mechanistic analyses assessed surface singular-value structure, vector covariance organization, post-deflation geometric motion, and duration-matched 13-dimensional state-space speed. Inference used patient-clustered robust standard errors with Holm correction. Results: LAD occlusion produced greater peak injury-surface burden than RCA (adjusted geometric-mean ratio 1.64, 95% CI 1.16-2.31) and LCx (1.77, 1.19-2.63), and greater peak eight-dimensional vector magnitude than RCA (1.65, 1.14-2.41) and LCx (1.90, 1.25-2.88). During occlusion, the surface was nearly rank-one (median first-component energy fraction 0.991), and vector covariance was dominated by a single mode (median PC1 variance fraction 0.940). After balloon deflation, median surface burden fell from 71.7 to 37.7, singular-value entropy increased, and mean alignment between the injury vector and the dominant covariance axis decreased (mean change -0.195, 95% CI -0.259 to -0.132). In a duration-matched comparison, median 13-dimensional state-space speed increased from 0.084 in the final 60 seconds of occlusion to 0.252 in the first 60 seconds after deflation (mean change +0.141, 95% CI +0.111 to +0.172). Polarity exhibited the most pronounced territory-specific trajectory, with LAD differing from both RCA and LCx during occlusion and recovery. Conclusions: Controlled coronary occlusion produces a coherent multidimensional ST injury state. Balloon deflation reduces injury amplitude while triggering rapid electrical reorganization rather than simple reversal. Coronary territory is expressed both in peak injury magnitude and, most distinctly, in polarity dynamics.

## Introduction

The 12-lead electrocardiogram (ECG) remains the fastest widely available representation of acute myocardial ischemia and is central to contemporary acute coronary syndrome pathways [1,2]. Clinical interpretation, however, necessarily reduces a high-dimensional signal to a small number of measurements, such as ST elevation in selected leads, summed ST deviation, or the degree of ST-segment resolution. These quantities are clinically important, and ST resolution has established prognostic value, but they do not explicitly describe how ischemic information is organized across leads, across the ST interval, or through time [3].

Controlled coronary balloon occlusion provides a rare human model in which the timing of both the onset and removal of a coronary occlusive stimulus is known. The STAFF program and the STAFF III database have shown that acute ischemia produces substantial lead-, time-, and patient-dependent changes in conventional ST segments, high-frequency QRS components, and vector-derived measurements [4-10]. Earlier multilead ST-segment fingerprint and continuous-monitoring studies also support the idea that distributed ST patterns carry information beyond isolated lead amplitudes [11,12]. More recent work has extended this concept toward spatiotemporal and vector representations rather than isolated ST amplitudes [13,14].

We therefore treated the ischemic ECG as an instantaneous multidimensional electrical state. Repeated ST measurements across precordial leads and fixed ST-time offsets form a lead-by-time surface, while a complementary multilead vector captures nonredundant spatial projections. These representations can then be tracked through sequential windows as a state trajectory. Throughout this report, "injury" denotes the classical electrocardiographic injury current or ST-deviation phenotype and does not imply irreversible myocardial necrosis.

The study addressed three linked questions. First, does peak multidimensional ST injury differ by occluded coronary territory? Second, does acute ischemia generate a coherent low-dimensional electrical state, and how does that organization change after balloon deflation? Third, is coronary territory encoded in the temporal evolution of that state? Peak injury magnitude was the focus of the primary physiological analysis, whereas low-rank organization and state dynamics were examined in mechanistic secondary analyses. Broader exploratory metrics were considered hypothesis-generating.

## Materials and methods

Study design and data source

We performed a secondary analysis of the de-identified STAFF III database distributed by PhysioNet (version 1.0.0; dataset DOI 10.13026/C20P4H) [4,15]. STAFF III was acquired in 1995-1996 at Charleston Area Medical Center during elective prolonged balloon angioplasty in the pre-stent era [4-6]. The recordings contain standard 12-lead ECGs before, during, and in many cases after balloon inflation. Precordial leads used standard chest positions, and limb leads were acquired in the Mason-Likar configuration. Signals were digitized at 1000 Hz with 0.625 microvolt amplitude resolution [4].

The released annotation spreadsheet contains 152 balloon occlusions in 104 patients and, when parsed directly, yields 58 right coronary artery (RCA), 58 left anterior descending (LAD), 33 left circumflex (LCx), and three left-main occlusions. Formal territory analyses were restricted to LAD, RCA, and LCx (149 occlusions in 102 patients). Artery assignments and inflation and deflation times were taken directly from the released annotation spreadsheet and preserved in the locked analysis dataset.

Separate baseline control recordings were used for all 152 occlusions according to a prespecified hierarchy: BC1 first, followed by BC2 and then BR when necessary. No dynamic trajectory failed extraction. Ninety recordings provided at least 180 seconds of post-deflation follow-up; shorter recordings were retained when valid and were handled by analysis-specific availability criteria. Because balloon deflation does not establish tissue-level reperfusion, the subsequent interval is termed post-deflation or recovery rather than physiological reperfusion.

This study analyzed de-identified, publicly available data and involved no new participant contact or intervention. The STAFF III database is available through PhysioNet [4,15]. The analyses were performed in Google Colaboratory using Python version 3.12 (Python Software Foundation, Wilmington, DE, USA). Figures were generated with Matplotlib version 3.10.0 (Matplotlib Development Team; https://matplotlib.org). The principal analytic Python scripts are publicly available at https://github.com/DumitrescuPatrick/staffiii-st-injury-state-dynamics.

ECG preprocessing and dynamic windows

Signals were filtered with a zero-phase 0.5-40 Hz Butterworth filter, and nonfinite samples were interpolated before analysis. Because high-pass filtering can influence absolute ST amplitudes, all ST measurements were interpreted as baseline-adjusted within-recording changes rather than absolute diagnostic ST elevations. R peaks were identified using a multilead consensus QRS detector applied to QRS-band-filtered signals with physiologic heart-rate limits and adaptive thresholds. Dynamic features were calculated in 10-second windows advanced every five seconds.

An exploratory, retrospective QRS-duration screening assessment was performed for the 149 analyzed occlusions using 12-second ECG excerpts obtained during early and late balloon inflation. One shorter occlusion contributed only a single excerpt. QRS-duration estimates were compared using all nine recorded leads and, separately, leads V1-V6 under three detection thresholds. An occlusion was considered screen-positive when estimates exceeded 100 milliseconds across all six settings in at least one examined excerpt. These automated measurements were not independently adjudicated by manual QRS-onset and QRS-offset measurements.

Dynamic ST injury surface and eight-dimensional ST injury vector

For each accepted beat, the PR reference level was calculated from R-100 to R-60 ms. The dynamic ST injury surface consisted of baseline-adjusted amplitudes in leads V1-V6 at seven fixed offsets (R+80, +100, +120, +140, +160, +180, and +200 ms), yielding a 6 x 7 matrix. R+80 denotes 80 ms after the detected R-wave peak. ST injury-surface burden was defined as the sum of absolute baseline-adjusted surface values multiplied by the 20-millisecond sampling interval. This metric is an integrated electrical burden across the sampled lead-time grid; it is not a literal geometric surface area and not an anatomical estimate of infarct volume.

A complementary eight-dimensional ST injury vector was formed from leads I, II, and V1-V6 using baseline-adjusted ST amplitude centered at R+120 ms with a ± 10 ms averaging interval. Vector magnitude provided an independent multidimensional measure of injury amplitude. Fixed R-referenced offsets were chosen for reproducibility within this dataset; J-point-anchored or QRS-duration-adaptive sampling remains an important external sensitivity analysis, particularly for wide-QRS recordings [10].

Low-rank organization and multidimensional state dynamics

Singular-value decomposition of each 6 x 7 surface quantified the fraction of surface energy captured by the first singular component and the singular-value entropy. For the eight-dimensional vector, covariance eigendecomposition quantified the fraction of variance explained by the first principal component (PC1) and the alignment between the mean injury vector and the dominant covariance axis. These measures were used to assess whether ischemia produces a coherent electrical pattern and whether that organization persists after balloon deflation.

For state-space analyses, the instantaneous 13-dimensional ST injury state comprised log-transformed surface burden, log-transformed vector magnitude, lead centroid, ST-offset centroid, covariance trace, tensor fractional anisotropy, normalized roughness, polarity index, normalized entropy, surface singular-value entropy, vector entropy, and the sine and cosine of twice the orientation angle. The coordinates were standardized using robust median and interquartile-range scaling before calculating state-space trajectory measures. State-space distances were calculated using the Euclidean norm of differences between consecutive standardized 13-dimensional states. No additional feature-specific weights were applied after median/IQR scaling. Robust scaling used $z = \frac{x - \mathrm{median}}{\mathrm{IQR}}$. The median (IQR) values for the 13 coordinates, in the order listed above, were 4.027037 (1.172430), 0.166190 (0.187952), 3.138886 (0.689704), 146.910030 (10.076289), 1.621313 (0.313870), 0.335524 (0.232825), 0.753301 (0.312932), −0.665372 (1.346193), 0.929367 (0.042286), 0.040601 (0.095764), 0.715061 (0.165560), −0.021954 (0.492640), and −0.965926 (0.265786), respectively. Sequential states were used to calculate path rate, mean and peak speed, acceleration, and related trajectory measures. To minimize bias arising from unequal observation durations, the principal dynamic sensitivity analysis compared the final 60 seconds of occlusion with the first 60 seconds after balloon deflation in the same 143 occlusions. Surface-centroid motion and tensor-orientation rotation were analyzed as complementary geometric measures.

**Table 1:** Definitions and scaling parameters of the 13-dimensional ST injury-state Robust scaling and weighting. For coordinate xⱼ, $z_j = \frac{x_j - \mathrm{median}_j}{\mathrm{IQR}_j}$; consecutive states use $d = \sqrt{\sum_{j=1}^{13}(z_{j,t+1} - z_{j,t})^2}$, with no additional feature-specific weights after standardization. Sᵢⱼ: baseline-adjusted ST-surface amplitude (six precordial leads × seven R-referenced offsets), vₖ: eight-lead ST-vector component, tⱼ: offset in milliseconds, C: 2D surface covariance tensor, λ₁, λ₂: tensor eigenvalues, θ: principal tensor orientation

Injury-state coordinate	Mathematical definition	Units before robust scaling	Median	IQR
1. state_log_surface_burden	Log-transformed surface burden: $B = 20 \sum_{i,j} |S_{ij}|$	dimensionless (B in mV·ms)	4.027037	1.172430
2. state_log_vector_magnitude	Log-transformed vector magnitude: $M = \sqrt{\sum_{k=1}^{8} v_k^2}$	dimensionless (M in mV)	0.166190	0.187952
3. centroid_lead	$C_L = \frac{\sum_{i,j} i|S_{ij}|}{\sum_{i,j}|S_{ij}|}$	lead index (1-6)	3.138886	0.689704
4. centroid_st_ms	$C_T = \frac{\sum_{i,j} t_j |S_{ij}|}{\sum_{i,j} |S_{ij}|}$	ms	146.910030	10.076289
5. cov_trace	$\operatorname{tr}(C) = C_{11} + C_{22} = \lambda_1 + \lambda_2$	squared tensor-axis units	1.621313	0.313870
6. tensor_fractional_anisotropy	Conventional 2D $\mathrm{FA} = \frac{|\lambda_1 - \lambda_2|}{\sqrt{\lambda_1^2 + \lambda_2^2}}$	dimensionless	0.335524	0.232825
7. normalized_roughness	$R_{\mathrm{norm}} = \frac{\displaystyle \sum_{i=1}^{5}\sum_{j=1}^{7}|S_{i+1,j}-S_{ij}|+\sum_{i=1}^{6}\sum_{j=1}^{6}|S_{i,j+1}-S_{ij}|}{\displaystyle \sum_{i=1}^{6}\sum_{j=1}^{7}|S_{ij}|}$	dimensionless	0.753301	0.312932
8. polarity_index	$P = \frac{\sum_{i,j} S_{ij}}{\sum_{i,j} |S_{ij}|}$	dimensionless	-0.665372	1.346193
9. normalized_entropy	$H = -\frac{\sum_{i,j} p_{ij}\ln(p_{ij})}{\ln(42)}; \quad p_{ij} = \frac{|S_{ij}|}{\sum_{i,j}|S_{ij}|}$	dimensionless	0.929367	0.042286
10. surface_svd_entropy	$H_{\mathrm{SVD}} = -\frac{\sum_{k} q_k \ln(q_k)}{\ln(6)}$	dimensionless	0.040601	0.095764
11. vector_entropy	$H_{\mathrm{vector}} = -\frac{\sum_{k=1}^{8} p_k \ln(p_k)}{\ln(8)}; \quad p_k = \frac{|v_k|}{\sum_{l=1}^{8}|v_l|}$	dimensionless	0.715061	0.165560
12. orientation_sin2	sin(2θ)	dimensionless	-0.021954	0.492640
13. orientation_cos2	cos(2θ)	dimensionless	-0.965926	0.265786

Territory-specific temporal analysis

Occlusion trajectories were normalized to 0%, 25%, 50%, 75%, and 100% of balloon-inflation duration. Recovery trajectories were evaluated at 0, 30, 60, 120, and 180 seconds after deflation in the strict complete-180-second cohort. Polarity was defined from the balance of positive and negative absolute surface mass and ranged from -1 to +1. Global artery-by-time models used categorical time and patient-clustered robust standard errors. The p-values for pairwise whole-trajectory interaction tests were adjusted using the Holm method across the three artery pairs.

Statistical analysis

Peak injury-surface burden and peak eight-dimensional vector magnitude were the primary physiological outcomes. Because both were right-skewed, log-transformed outcomes were modeled as a function of artery group and inflation duration, centered and scaled per 100 seconds, with cluster-robust standard errors at the patient level. Results are reported as adjusted geometric-mean ratios with 95% confidence intervals (CIs). Pairwise p-values were corrected by the Holm method. A 2,000-replicate patient-cluster bootstrap assessed the stability of the primary artery contrasts.

Mechanistic occlusion-versus-post-deflation comparisons used patient-clustered inference with Holm adjustment within related metric families. Longitudinal artery-by-time analyses used categorical checkpoint models with patient-clustered standard errors. The strict 180-second recovery and matched-duration analyses were sensitivity analyses designed to address unequal post-deflation follow-up. The broader tensor, hysteresis, and nested territory-classification analyses were exploratory and are not used as primary evidence in this report. Two-sided p < 0.05 after the specified multiplicity correction was considered statistically significant.

## Results

All 152 balloon occlusions were successfully processed, and a dynamic trajectory was extracted for each occlusion. The principal LAD, RCA, and LCx cohort comprised 149 occlusions in 102 patients. Ninety recordings had at least 180 seconds of post-deflation follow-up, and the strict complete-180-second longitudinal cohort included 88 occlusions in 71 patients. The duration-matched 60-second state-dynamics analysis included 143 occlusions in 99 patients (Table 2).

**Table 2:** STAFF III study cohort and analysis sets The primary analysis cohort excluded left main occlusions because of the small sample size and mixed coronary territory involvement. LAD: left anterior descending artery, RCA: right coronary artery, LCx: left circumflex artery, LM: left main, IQR: interquartile range

Characteristic	Value
Patients, n	104
Balloon occlusions, n	152
RCA occlusions, n	58
LAD occlusions, n	58
LCx occlusions, n	33
LM occlusions, n	3
Primary analysis cohort (LAD, RCA, and LCx)	149 occlusions/102 patients
Inflation duration, median (IQR), s	290 (181-300)
Dynamic trajectories successfully extracted	152/152
Recordings with ≥180 s post-deflation follow-up	90
Strict complete 180-s recovery cohort	88 occlusions/71 patients
Matched 60-s state-dynamics cohort	143 occlusions/99 patients

Peak multidimensional ST injury differed by occluded coronary artery

Peak ST injury-surface burden differed across LAD, RCA, and LCx (global p = 0.0046). LAD occlusion produced 1.64-fold greater adjusted peak burden than RCA (95% CI 1.16-2.31; Holm p = 0.0139) and 1.77-fold greater burden than LCx (95% CI 1.19-2.63; Holm p = 0.0139); RCA and LCx did not differ. Peak eight-dimensional vector magnitude showed the same pattern (global p = 0.0046): LAD exceeded RCA by 1.65-fold (95% CI 1.14-2.41; Holm p = 0.0170) and LCx by 1.90-fold (95% CI 1.25-2.88; Holm p = 0.0075). Patient-cluster bootstrap estimates were nearly identical to the primary estimates and supported both LAD contrasts (Figure 1).

**Figure 1:**
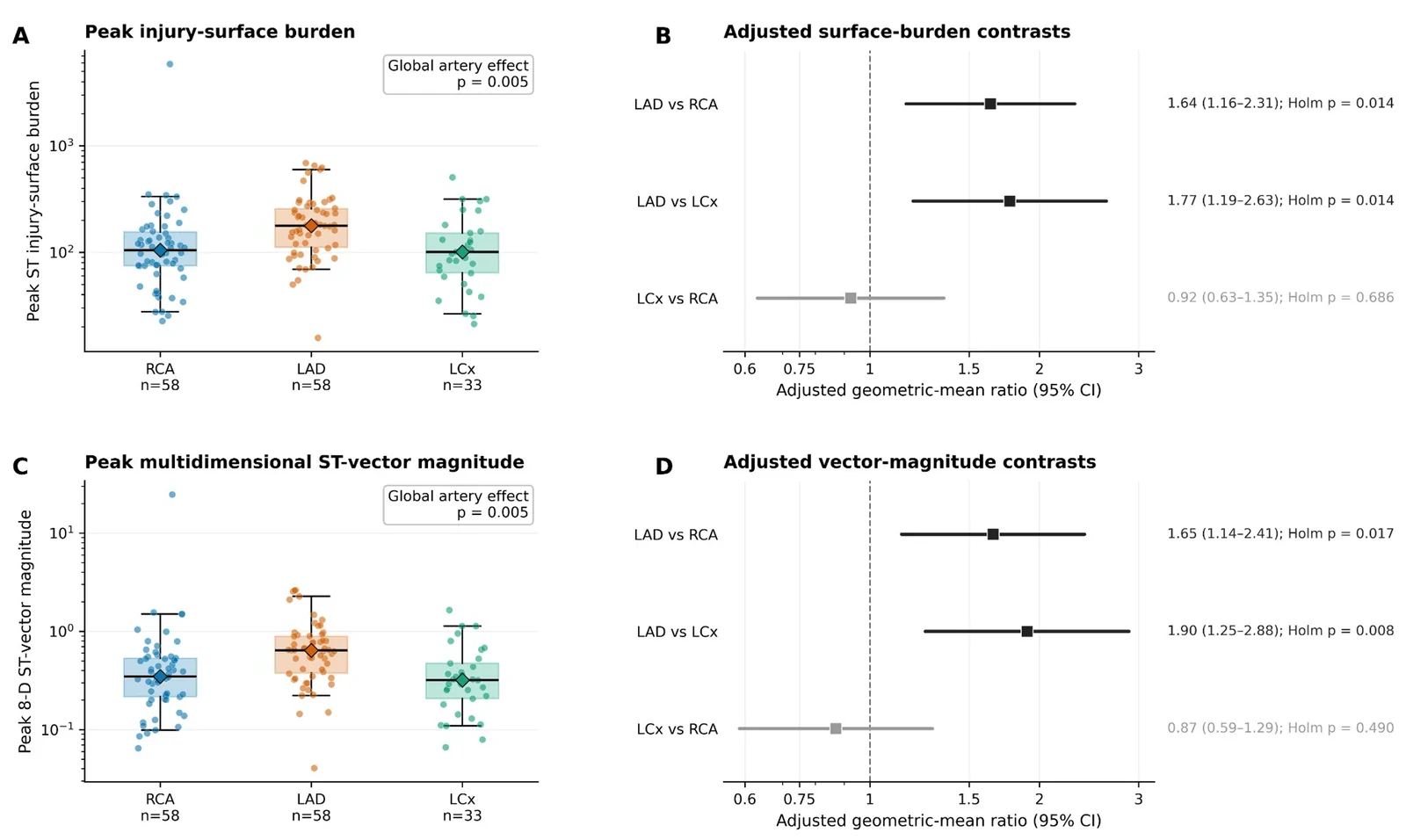
Peak multidimensional ST injury by occluded coronary artery The primary three-territory cohort comprised 149 balloon occlusions in 102 patients (RCA n = 58, LAD n = 58, LCx n = 33); the three left-main occlusions were excluded from formal territory comparisons. Individual occlusions are shown for peak ST injury-surface burden and peak eight-dimensional ST injury vector magnitude. Forest plots show adjusted geometric-mean ratios and 95% CIs from log-linear models with patient-clustered robust standard errors and adjustment for inflation duration. Pairwise p-values are Holm-adjusted. RCA: right coronary artery, LAD: left anterior descending artery, LCx: left circumflex artery, CIs: confidence intervals

Acute ischemia formed a highly coherent electrical state

During balloon occlusion, the precordial ST injury surface was nearly rank-one, with a median first-component energy fraction of 0.991. The eight-dimensional vector covariance was similarly dominated by a single mode, with a median PC1 variance fraction of 0.940. These concordant surface and vector findings indicate that acute ischemia organizes the multilead ST response into a strongly coordinated electrical state rather than a collection of independent lead-by-time abnormalities (Figure 2).

**Figure 2:**
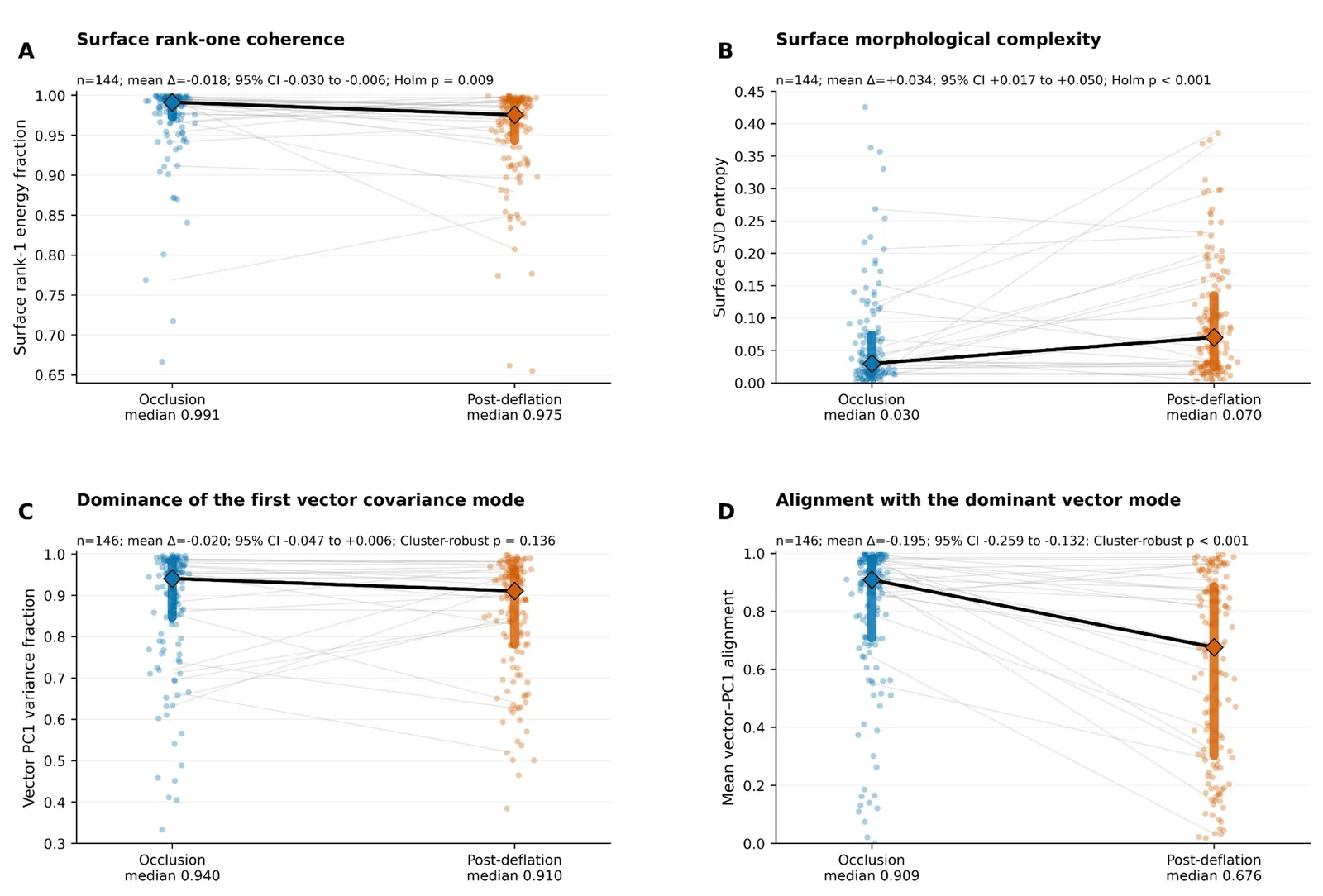
Low-dimensional organization during occlusion and post-deflation reorganization Surface singular-value decomposition paired analyses included 144 occlusions from 99 patients. The descriptive vector panels display 146 occlusions with paired occlusion and post-deflation measurements; the patient-clustered inferential statistics shown for vector metrics use the prespecified LAD, RCA, and LCx cohort of 143 occlusions from 99 patients, excluding the three left-main occlusions. All observations are shown, with a representative subset of paired lines for visual clarity. PC1: first principal component, LAD: left anterior descending, RCA: right coronary artery, LCx: left circumflex, CI: confidence interval

After balloon deflation, the first-component energy fraction decreased to a median of 0.975 (mean change -0.0178, 95% CI -0.0295 to -0.0060; Holm p = 0.009), while singular-value entropy increased from 0.030 to 0.070 (mean change +0.0339, 95% CI +0.0174 to +0.0503; Holm p < 0.001). The PC1 variance fraction of the ST injury vector did not change significantly, but alignment between the mean injury vector and the dominant covariance axis decreased from a median of 0.909 to 0.676 (mean change -0.195, 95% CI -0.259 to -0.132; patient-clustered p < 0.001). Post-deflation reorganization was therefore characterized primarily by a loss of coherent alignment rather than a uniform increase in vector covariance dimensionality.

Balloon deflation reduced injury magnitude but accelerated state reorganization

Median ST injury-surface burden fell from 71.7 during occlusion to 37.7 after balloon deflation (mean paired change -48.7, 95% CI -66.4 to -30.9; Holm p < 0.001). At the same time, surface-centroid mean speed increased from a median of 0.015 to 0.032, and tensor-orientation rotation rate increased from 0.607 to 1.357 degrees per second. Thus, the reduction in injury amplitude did not indicate that the electrical state had become static.

The duration-matched state-space analysis provided the strongest temporal sensitivity test. Median 13-dimensional state-space speed increased from 0.084 during the final 60 seconds of occlusion to 0.252 during the first 60 seconds after deflation, with a mean increase of +0.141 (95% CI +0.111 to +0.172; Holm p < 0.001). Path rate and peak speed increased similarly. Fixed-horizon analyses additionally showed increasing return-path mismatch over 180 seconds, supporting partial, non-retracing recovery rather than simple reversal of the ischemic trajectory (Figure 3).

**Figure 3:**
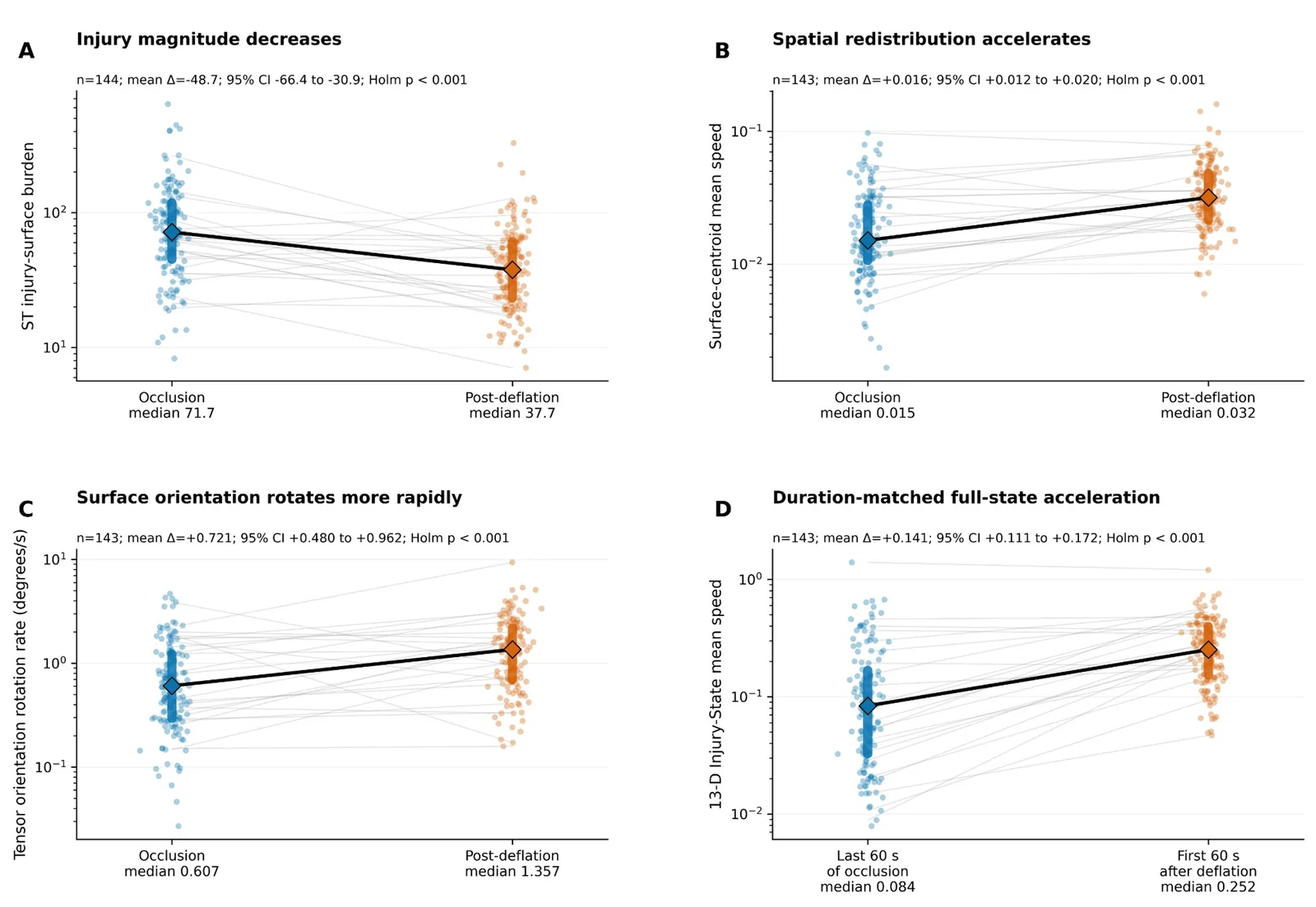
Injury reduction with accelerated state reorganization after balloon deflation Panel A includes 144 paired occlusions from 99 patients for ST injury-surface burden. Panels B and C include 143 paired occlusions from 99 patients for surface-centroid mean speed and tensor-orientation rotation rate. Panel D includes 143 occlusions from 99 patients in the duration-matched comparison of the final 60 seconds of occlusion with the first 60 seconds after deflation. All observations are shown, with representative paired lines for visual clarity.

Polarity was the dominant territory-specific temporal signature

Polarity distinguished the LAD territory from the RCA and LCx territories more clearly than any other trajectory feature. During normalized occlusion, the artery-by-time interaction remained significant after multiplicity correction (Holm p < 0.001). LAD polarity shifted from strongly negative values at onset to strongly positive values by 25% of the occlusion duration and remained positive thereafter, whereas RCA and LCx polarity remained predominantly negative. Whole-trajectory tests confirmed LAD-versus-RCA and LAD-versus-LCx differences (both Holm p < 0.001), with no LCx-versus-RCA difference.

The distinction persisted after balloon deflation in the strict complete-180-second cohort (artery-by-time Holm p = 0.007). LAD underwent a pronounced polarity reversal toward negative values and differed from RCA (Holm p = 0.003) and LCx (Holm p = 0.004), whereas LCx and RCA trajectories were nearly superimposable. Polarity was therefore the most reproducible territory-specific dynamic feature in the present framework (Figure 4). Key primary and mechanistic results are summarized in Table 3.

**Figure 4:**
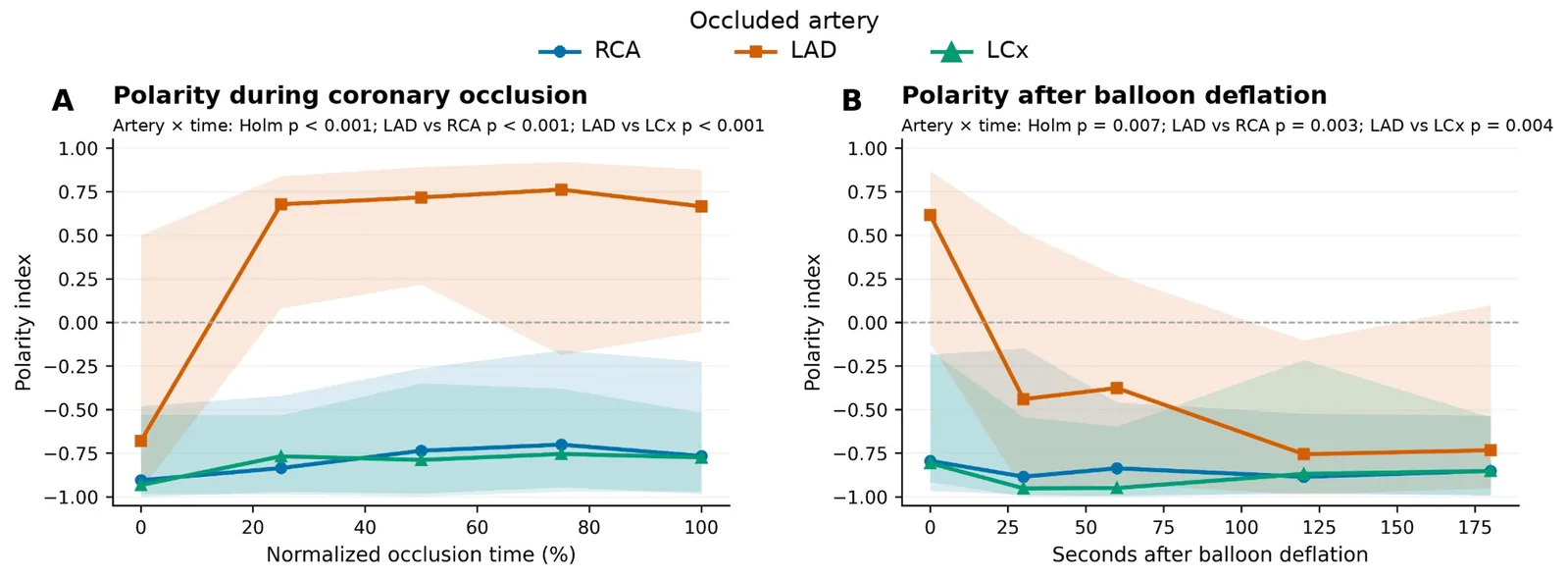
Polarity is the dominant territory-specific temporal signature During occlusion, descriptive trajectories include all 149 LAD, RCA, and LCx occlusions. The formal artery-by-time model used 144 occlusions from 100 patients with complete checkpoint data. The strict complete-180-second recovery cohort comprised 88 occlusions from 71 patients. Curves show medians and interquartile ranges. Pairwise p-values are Holm-adjusted whole-trajectory interaction tests. LAD: left anterior descending, RCA: right coronary artery, LCx: left circumflex

**Table 3:** Key primary, mechanistic, and longitudinal findings Primary contrasts are adjusted geometric-mean ratios with 95% CIs. Mechanistic rows summarize median state values and mean paired differences with 95% CIs. Longitudinal rows summarize global artery-by-time interaction tests. The vector-PC1 alignment row has been matched to the Figure 2D medians. CIs: confidence intervals, LAD: left anterior descending artery, LCx: left circumflex artery, PC1: first principal component, RCA: right coronary artery, ST: ST segment, SVD: singular-value decomposition

Analysis domain	Endpoint or contrast	Effect estimate	Adjusted p-value
Primary	Peak injury-surface burden, LAD vs RCA	Adjusted geometric-mean ratio 1.64 (95% CI, 1.16-2.31)	0.014
Primary	Peak injury-surface burden, LAD vs LCx	Adjusted geometric-mean ratio 1.77 (95% CI, 1.19-2.63)	0.014
Primary	Peak eight-dimensional ST injury vector magnitude, LAD vs RCA	Adjusted geometric-mean ratio 1.65 (95% CI, 1.14-2.41)	0.017
Primary	Peak eight-dimensional ST injury vector magnitude, LAD vs LCx	Adjusted geometric-mean ratio 1.90 (95% CI, 1.25-2.88)	0.008
Mechanistic	First-component energy fraction, occlusion to post-deflation	Median 0.991 to 0.975; mean difference -0.018 (95% CI, -0.030 to -0.006)	0.009
Mechanistic	Surface SVD entropy, occlusion to post-deflation	Median 0.030 to 0.070; mean difference +0.034 (95% CI, +0.017 to +0.050)	<0.001
Mechanistic	Mean injury-vector alignment with the dominant covariance axis, occlusion to post-deflation	Median 0.909 to 0.676; mean difference -0.195 (95% CI, -0.259 to -0.132)	<0.001
Mechanistic	Mean 13-dimensional state-space speed, final 60 s of occlusion to first 60 s post-deflation	Median 0.084 to 0.252; mean difference +0.141 (95% CI, +0.111 to +0.172)	<0.001
Longitudinal	Polarity artery-by-time during occlusion	Global artery-by-time interaction	<0.001
Longitudinal	Polarity artery-by-time after balloon deflation	Global artery-by-time interaction	0.007

Retrospective automated QRS-duration screening identified 11 of 149 coronary occlusions (7.4%) with estimates consistently exceeding 100 milliseconds across the tested measurement settings.

## Discussion

This study supports a compact physiological model of the ischemic ECG: controlled coronary occlusion creates a coherent multidimensional ST injury state, and balloon deflation decreases the magnitude of that state while accelerating its reorganization. Three principal findings support this interpretation. First, LAD occlusion generated the largest peak multidimensional injury response. Second, during occlusion, both the lead-by-ST-time surface and the independent multilead vector were dominated by a single organizing mode. Third, post-deflation recovery did not simply retrace the ischemic trajectory: injury amplitude decreased while surface complexity, surface-centroid motion, and state-space speed increased. Polarity provided the clearest territory-specific temporal signature.

Ischemic ST response is coherent rather than fragmented

The median first-component energy fraction of approximately 99% is notable because the surface contains 42 baseline-adjusted measurements. A single component therefore captures most of the joint lead-by-time structure during controlled ischemia. The parallel dominance of the first principal component of vector covariance provides an independent representation of the same phenomenon. Prior STAFF work established that conventional ST deviation, high-frequency QRS behavior, and vector-derived quantities each respond to acute ischemia [5-10]. The present analysis extends these findings and earlier concepts of multilead ST-segment fingerprinting and continuous monitoring [11,12] by demonstrating that the multilead ST response is not merely large in magnitude but also highly organized.

However, low-rank organization should not be interpreted as evidence of physiological simplicity. The instantaneous surface can be coherent, while the integrated injury state still evolves across multiple dimensions. The distinction becomes particularly clear after deflation: surface rank-one dominance decreases, singular-value entropy rises, and the mean injury vector becomes much less aligned with the dominant covariance axis even though PC1 continues to explain a large fraction of vector variance. The most defensible interpretation is reorganization or decoherence of the injury state, not a blanket claim that every covariance measure becomes more multidimensional.

Recovery is dynamic, not merely smaller

Conventional recovery metrics emphasize reduction of ST deviation, and ST-segment resolution remains an established marker of myocardial reperfusion and prognosis [3]. Our findings do not challenge that principle but provide an additional perspective on electrical recovery. In this controlled model, decreasing injury amplitude was accompanied by faster surface-centroid motion, a higher tensor-orientation rotation rate, increasing surface complexity, and an approximately threefold increase in median state-space speed in the duration-matched comparison. Early post-deflation ECG evolution therefore provides information about the trajectory and rate of recovery, in addition to the magnitude of residual ST deviation.

Because balloon deflation is not synonymous with complete tissue-level reperfusion, these observations should not be interpreted as a direct description of clinical reperfusion injury. Rather, they identify an electrical transition immediately after removal of a precisely timed occlusive stimulus. Reactive hyperemia, extracellular potassium washout, and regional recovery of membrane potentials may contribute to the rapid electrical-state reorganization observed after balloon deflation, although these mechanisms were not directly measured. External validation in ST-elevation myocardial infarction and percutaneous coronary intervention cohorts is required to determine whether analogous trajectory features track epicardial flow, microvascular reperfusion, infarct size, or clinical outcome.

Territory is encoded in magnitude and orientation

LAD occlusion produced greater peak surface burden and vector magnitude than RCA or LCx, but coronary territory explained only a modest fraction of total inter-occlusion variability. Polarity was a stronger discriminator of coronary territory over time. LAD showed a rapid positive transition during occlusion and a marked negative reversal after deflation, whereas RCA and LCx remained predominantly negative and were difficult to distinguish from one another. This pattern is consistent with the territory-dependent orientation of injury currents and supports preserving sign and direction rather than relying exclusively on unsigned amplitude summaries [9,14].

Recent cardiac magnetic resonance work has also cautioned that conventional ECG localization and simplified ST-axis orientation correspond imperfectly to the anatomical distribution of infarction, while global ST burden may better reflect injury extent [16]. That observation is consistent with our findings: peak burden and vector measures should be interpreted as indicators of electrical injury magnitude rather than direct measures of anatomical infarct volume, whereas polarity should be interpreted as an electrical phenotype of territory-dependent orientation rather than a precise anatomical locator.

Clinical and methodological implications

The principal contribution of this work is not a culprit-artery classifier. It is a representation that simultaneously preserves three properties of the ischemic ECG: magnitude, organization, and trajectory. Exploratory nested patient-grouped models did show greater territory discrimination for the full injury state than for conventional ST amplitudes, but these internally validated results were treated as hypothesis-generating and not as primary evidence. A critical next step is prospective external validation in cohorts of patients with acute ST-elevation myocardial infarction or undergoing percutaneous coronary intervention, incorporating angiographic flow, biomarkers, cardiac magnetic resonance imaging, ventricular function, and clinical outcomes.

Limitations

Several limitations merit emphasis. First, STAFF III is a controlled elective balloon-occlusion model from the pre-stent era and differs from spontaneous plaque rupture, thrombus, collateral flow, and contemporary reperfusion care. Second, post-deflation recordings varied in duration; complete 180-second, fixed-horizon, and duration-matched analyses were used to address this. Third, three left-main occlusions were insufficient for formal analysis. Fourth, fixed R-referenced ST offsets may sample late QRS activity in wide-QRS recordings, and J-point-anchored sensitivity analysis is warranted [10]. Fifth, many geometric and trajectory variables were exploratory, so the main analysis restricts inference to a small set of linked hypotheses and multiplicity-controlled results. Sixth, the dataset contains repeated occlusions in some patients, which motivated patient-clustered inference throughout. Additionally, no external clinical cohort was available, and the state-space representation requires independent validation before clinical use. Because automated QRS-duration estimates were not independently adjudicated by manual QRS-onset and QRS-offset measurements, potential inclusion of terminal ventricular depolarization at the fixed R+80 ms ST-sampling point cannot be excluded. Mason-Likar torso placement of the limb electrodes may alter measured amplitudes and electrical axes relative to conventional diagnostic limb-lead placement, limiting direct generalization of the vector findings. The absence of posterior and right-sided chest leads limits assessment of posterior and right ventricular electrical injury, while the lack of simultaneous microvascular perfusion measurements prevents direct confirmation of tissue-level reperfusion.

## Conclusions

Controlled human coronary occlusion produces a coherent multidimensional ST injury state. LAD occlusion generates the greatest peak multidimensional injury magnitude, while the ischemic surface and vector exhibit predominantly low-dimensional organization. Balloon deflation reduces injury amplitude but accelerates electrical-state reorganization, and polarity provides a distinctive LAD temporal signature. These findings motivate further investigation of dynamic ECG representations that preserve the magnitude, organization, and trajectory of acute ischemic electrical injury. However, their incremental clinical value remains to be established through independent validation.
